# Genomic Estimated Breeding Values Using Genomic Relationship Matrices in a Cloned Population of Loblolly Pine

**DOI:** 10.1534/g3.113.005975

**Published:** 2013-05-01

**Authors:** Jaime Zapata-Valenzuela, Ross W. Whetten, David Neale, Steve McKeand, Fikret Isik

**Affiliations:** * Department of Forestry and Environmental Resources, North Carolina State University, Raleigh, North Carolina 27695; † Department of Plant Science, University of California-Davis, Davis, California 95616

**Keywords:** GenPred, Shared data resource, Pinus taeda, quantitative genetics, best linear unbiased prediction

## Abstract

Replacement of the average numerator relationship matrix derived from the pedigree with the realized genomic relationship matrix based on DNA markers might be an attractive strategy in forest tree breeding for predictions of genetic merit. We used genotypes from 3461 single-nucleotide polymorphism loci to estimate genomic relationships for a population of 165 loblolly pine (*Pinus taeda* L.) individuals. Phenotypes of the 165 individuals were obtained from clonally replicated field trials and were used to estimate breeding values for growth (stem volume). Two alternative methods, based on allele frequencies or regression, were used to generate the genomic relationship matrices. The accuracies of genomic estimated breeding values based on the genomic relationship matrices and breeding values estimated based on the average numerator relationship matrix were compared. On average, the accuracy of predictions based on genomic relationships ranged between 0.37 and 0.74 depending on the validation method. We did not detect differences in the accuracy of predictions based on genomic relationship matrices estimated by two different methods. Using genomic relationship matrices allowed modeling of Mendelian segregation within full-sib families, an important advantage over a traditional genetic evaluation system based on pedigree. We conclude that estimation of genomic relationships could be a powerful tool in forest tree breeding because it accurately accounts both for genetic relationships among individuals and for nuisance effects such as location and replicate effects, and makes more accurate selection possible within full-sib crosses.

The expected additive genetic relationships (genetic covariances) derived from a pedigree are based on probabilities that gene pairs are identical by descent (IBD) (Lynch and Walsh 1998). For example, the expected average genetic covariance between full-sibs is 0.5 because two individuals with the same parents are expected to share 50% of their alleles IBD. Pedigree-based additive genetic relationships have been widely used to estimate genetic covariances and genetic merit of individuals in animal- and plant-improvement programs (Henderson 1975). However, genetic relationships derived from pedigrees ignore the random sampling of the two possible alleles from each parent at each locus during meiosis; this variation is defined as the Mendelian sampling term (Avendano *et al.* 2005). Traditional genetic evaluations based on pedigree do not trace individual alleles (VanRaden 2008).

van Arendonk *et al.* (1994) suggested that large numbers of DNA markers covering the genome could measure genetic similarity more accurately than a pedigree-based relationship matrix because the realized genetic covariances would be based on the actual proportion of the genome that is IBD between any two individuals. DNA markers can trace alleles that are IBD as well as alleles that are identical in state (VanRaden 2008). Genomic covariances are based on the fraction of total DNA that two individuals share (Stranden and Garrick 2009). In the classic “infinitesimal model” of quantitative genetics, genetic merit is considered to be the sum of thousands of allelic effects. In real genomes, those alleles are physically located at individual locations on the genome whose transmission can be traced through genetic markers. Dense markers can be used to trace IBD at each locus, and these IBD probabilities can then be used to construct relationship matrices (Forni *et al.* 2011).

With advancement in high-throughput genotyping technologies, realized genomic relationships are being used in cattle-breeding programs to increase selection efficiency (Goddard and Hayes 2007). Selection based on realized genomic relationships can produce more accurate predictions than the pedigree-based method because genomic selection can exploit variation created by Mendelian segregation during gamete formation. In simulation studies, the accuracy of prediction of net merit for young bulls was 63% compared with 32% when traditional pedigree-based relationships were used (VanRaden 2008). In another simulation by Villanueva *et al.* (2005), accuracies of predictions based on a realized genomic relationship matrix were greater than accuracies of predictions based on pedigree and phenotype. Such methods do not require known location of markers in the genome or estimation of relative effects of individual QTL on the trait.

Forest tree breeding programs are logistically difficult and expensive to carry out. It takes many years (typically 15 years or more for conifers) to complete a breeding cycle. For example, one loblolly pine (*Pinus taeda* L.) breeding program in the southern United States has completed its third cycle of breeding in 55 years (McKeand and Bridgwater 1998). One of the challenges tree breeding has experienced is tracking pedigrees created via controlled pollination, due to the cost of making large numbers of single-pair matings to maximize the likelihood of crossing high-value individuals to each other. Pollinating a group of female trees with mixed pollen and then constructing full pedigrees using DNA markers was suggested as an alternative way to reduce the cost but also track full pedigrees (Lambeth *et al.* 2001). El-Kassaby and Lstiburek (2009) extended the idea as a low-cost breeding option. Paternal parents of offspring were delineated for a *Larix occidentalis* population to reconstruct full pedigree information using SSR markers (El-Kassaby *et al.* 2011). However, fingerprinting and parentage analysis using DNA markers is conceptually not different from using a numerator relationship matrix generated from a known pedigree. The genetic relations between relatives are still categorical, not continuous, as are the expected additive genetic covariances derived from the reconstructed pedigree. Using DNA markers to construct realized genomic relationships for genetic evaluations in conifers is conceptually different. The major difference between realized genomic relationships and relationships based on pedigree reconstruction is that the realized genomic relationships predict the actual fraction of total DNA that two individuals share and utilize this information in predictions of genetic merit of individuals without phenotype.

In this study, using a small empirical data set, we tested the utility of genetic evaluation using genomic relationship matrices in a small population of loblolly pine and compared the reliability of estimates of the genetic merit of cloned pine individuals with those obtained by traditional pedigree-based genetic evaluation. The objectives of this study were to test two specific hypotheses: (1) the use of genomic relationships based on single-nucleotide polymorphism (SNP) markers to predict genetic merit is as efficient as traditional pedigree-based and phenotype genetic evaluation; and (2) genomic relationship matrices calculated by two different methods, the allele frequency and regression approaches suggested by Forni *et al.* (2011), do not differ in accuracies of predictions.

## Materials and Methods

### Phenotypic and genotypic data

Thirteen loblolly pine parents were used as females and males to generate nine full-sib families (hereafter family). The majority of full-sib families were obtained by single-pair mating design. Several full-sib families were genetically related by a common male or female parent (Zapata-Valenzuela *et al.* 2012). The number of progeny per family ranged from 3 to 37, and they were cloned via somatic embryogenesis (Bettinger *et al.* 2009). A total of 165 cloned full-sib progeny from nine crosses were field tested on 16 sites planted between 2000 and 2002. An alpha-lattice incomplete block design with single tree plots was used as the field layout. The test sites were located across varying edaphic conditions and productivity classes in the Coastal Plains of South Carolina, Georgia, and in the Gulf Coast of Mississippi in the southern United States. A total of 6253 pine trees were measured 5 years after they were planted. Height and diameter at 1.4 m above ground were assessed and used to calculate volume of trees according to Goebel and Warner (1966).

For the genotypic information, SNP markers based on 7535 resequenced amplicons were developed for loblolly pine by the Conifer Translational Genomics Network by integrating the results of the Allele Discovery of Economic Pine Traits project (Eckert *et al.* 2010). For marker analysis, we collected needles from 165 pine clones during the growing season in 2008. Samples were dried and shipped to University of California Davis for DNA extraction. Genotypes were obtained for a total of 5379 SNP markers using an Infinium SNP array, through a service provided by Illumina. We carried out exploratory data analysis on SNP markers using the ALLELE procedure of SAS GENETICS software (SAS Institute Inc. 2010). Of 5379 SNP markers analyzed, 1700 SNP markers were monomorphic (homozygous in all individuals), and 218 contained missing genotypes for more than 15% of the clones. Thus, a total of 3461 SNP markers were informative and used for calculations of realized genomic relationships. Phenotypic and genotypic data are provided in Supporting Information, File S1 and File S2.

### Imputation of missing genotypes

Matrix-based analytical methods require datasets with no missing values, but genotypes were missing for 1.2% of the total data points in a 165 pine trees by 3461 genotypes data matrix. We tested the effect of different imputation approaches by comparing the accuracy of breeding value predictions based on datasets with missing values imputed by different methods. Imputation methods were (1) allele frequencies, (2) replacing missing genotype by the major homozygote of the loci, and (3) using allele content as suggested by Gengler *et al.* (2007). The allele frequency method is a stochastic approach that imputes a categorical genotype (0, 1, or 2 copies of the minor allele), based on the frequency of all genotypes observed at the same locus, across all families. Across all missing values and loci, this approach should on average impute values that do not change the genotype frequencies observed in the nonmissing data. The method of Gengler *et al.* (2007) for imputation of missing genotypes is based on the idea that the covariance between genotypes is proportional to the additive genetic relationship between individuals. Genetic covariance (covariance of genetic values) arises because two related individuals have alleles that are IBD. The solutions of mixed model equations are predicted SNP genotypes for individuals. The solutions were continuous numeric values, centered on 1, because the gene content number could be 0, 1, or 2. Gene content for a biallelic locus cannot be less than 0 or greater than 2, so we scaled the values, adjusted by mean and SE, and truncated data to a range between 0 and 2 to keep gene content predictions in a realistic range of values. For the comparison of imputation methods, the accuracy (*r*) of the genomic estimated breeding value (GEBV) was calculated as:

$$r_{GEBV}=\sqrt{1-\frac{SE^{2}}{\left(1+f\right)\;\sigma_{a}^{2}}}$$ (1)

where SE is the standard error reported with the predicted breeding value of a clone. The term σ^2^_a_ is the estimated genetic variance, *f* is the inbreeding level, and 1+*f* is derived from the diagonal of numerator relationship matrix. For simplicity, the inbreeding coefficient was assumed to be zero for unrelated individuals.

### Traditional genetic evaluation using pedigree (best linear unbiased prediction based on pedigree, or ABLUP)

Traditional genetic evaluation relies on the inverse of genetic relationship matrix (**A^−^**^1^) derived from pedigree and phenotype. The model in matrix form is as follows

Y=Xb+Zu+e (2)

where **y** is the vector of observations representing the trait of interest (dependent variable), **X** and **Z** are the design or incidence matrices for the vectors of parameters **b** and **u**, respectively, which are the fixed covariates (*e.g.*, location effect) and random effects (tree) to be estimated, respectively. The term **u** is the vector of breeding values of trees with Var (**u**) = **A**σ^2^_a_, where **A** is the numerator relationship matrix and σ^2^_a_ is the additive genetic variance. The variance-covariance matrix of random effects (trees) in the linear mixed model is replaced by the **A** matrix to predict breeding values. The term **e** is the residual component or vector of residuals with Var (**e**) = **I**σ^2^_e_ = **R**, where **I** is the identity matrix and σ^2^_e_ is the error variance (Lynch and Walsh 1998; Mrode 2005). The predicted breeding values for all 165 cloned pine trees, using all available phenotypic data in the model, were denoted as estimated breeding value 1 (EBV1). The estimated breeding values for the cloned pine trees used in cross-validation, estimated without using the corresponding phenotypic data, were denoted as EBV2, following the same model of Eq. 2.

### Estimation of realized genomic relationships

We used the allele frequency and the regression methods to obtain genomic relationship matrices, as described by Legarra *et al.* (2009) and Forni *et al.* (2011). For all genotyped trees, in the allele frequency method we first standardized the variance of **u** as Var (**u**) = **ZZ***^T^* σ^2^_m_ = (**ZZ***^T^*/ [2 Σ_i_p_i_ (1 − p_i_)]) σ^2^_a_ = **G**σ^2^_a_, where the expression [2 Σ_i_p_i_ (1 − p_i_)] is twice the sum of heterozygosity of the markers, *p_i_* is allele frequency at locus *i*, **ZZ***^T^* represents the number of shared SNP alleles among two individuals, **Z***^T^* is the transpose of the **Z** matrix, σ^2^_m_ is the variance explained by markers, and σ^2^_a_ is the additive genetic variance. The division of **ZZ***^T^* by 2Σ_i_p_i_ (1 − p_i_) scales **G** to be analogous to the **A** matrix, which enables computation of the genomic relationships (VanRaden 2008). The genomic inbreeding coefficient for individual *j* is simply G_jj_ − 1, and genomic relationships between individuals *j* and *k* are obtained by dividing elements G_jk_ by square roots of diagonals G_jj_ and G_kk_ (VanRaden 2007, 2008).

The regression method does not require allele frequencies to obtain **G**, the realized genomic relationship matrix. The **G** matrix was produced by adjustment of mean homozygosity by regressing **MM***^T^* as a dependent variable on **A** as an independent variable to obtain **G**, as **MM***^T^* = g_o_**11***^T^* + g_1_**A** + **e**, where g_o_ is the intercept, g_1_ is the slope of the regression model, and **e** includes differences between the true value and the expected fraction of alleles shared in common. Thus, the expected value of **G** would be the numerator relationship matrix derived from pedigree (**A**) plus a constant matrix (*E* (**G**) = **A** + constant). It is simpler to do an extension of single marker association analysis to multiple marker regression to account for all genes (markers) simultaneously (Stranden and Garrick 2009). The regression system was written with summation notations, as in VanRaden (2008):

$$\left[\begin{array}{cc} n^{2} & \underset{j}{\sum }\underset{k}{\sum }A_{jk}\\ \underset{k}{\sum }\underset{j}{\sum }A_{jk} & \underset{j}{\sum }\underset{k}{\sum }A_{jk}^{2} \end{array}\right]\left[\begin{array}{c} {\hat{g}}_{0}\\ {\hat{g}}_{1} \end{array}\right]=\left[\begin{array}{c} \underset{j}{\sum }\underset{k}{\sum }{\left(MM^{T}\right)}_{jk}\\ \underset{j}{\sum }\underset{k}{\sum }{\left(MM^{T}\right)}_{jk}A_{jk} \end{array}\right]$$ (3)

In general, this method did not give singularities because the number of markers *m* was much larger than *n*, the number of individuals, which was required to obtain **G**^-1^. The **G** matrix may be singular, for example, if the number of markers *m* does not exceed the number of *n* individuals genotyped (VanRaden 2008).

### Genomic estimated breeding values (genomic best linear unbiased prediction, or GBLUP)

Genomic estimated breeding values were obtained using the following mixed model and substituting the **A** matrix by the two **G** matrices estimated using the alternative methods.

y=Xb+Za+e (4)

where **Z** is the incidence matrix of markers, **a** is the vector of marker effects, and **X**, **b**, and **e** are as explained in Eq. 2. The expected variance of vector **a** is Var(**a**) = **I**σ^2^_m_, where σ^2^_m_ is the variance explained by markers, and **I** is the identity matrix. The important difference from model 1 is that we let the sum **Za** across all marker loci (*m*) to be equal to the vector of breeding values. In other words, **u** = **Za**. The **A** matrix was replaced by the genomic relationship matrix **G** derived from allele frequencies as suggested by VanRaden (2008). The selection index equations were used to predict genetic merit **u** as follows:

$$\text{GEBV}(\hat{u})\;={G\;[G+R\lambda ]}^{-1}y\;-\;X\hat{b})$$ (5)

where λ = σ^2^_e_ /σ^2^_a_, the shrinkage factor in BLUP. The predicted value of **u** is the sum **Za** over all alleles that the individual inherited. We denoted the genomic estimated breeding values based on allele frequency method as GEBVa.

Using the **G** matrix derived from markers by the regression method, the estimated genetic merits of trees were obtained as:

$$\text{GEBV}(\hat{u})={{[R}^{-1}{+G}^{-1}\lambda ]}^{-1}{\text{R}}^{-1}(y-X\hat{b})$$ (6)

We denoted the genomic estimated breeding values using this method as GEBVb. This method was more efficient than the method given in Eq. 5 because **G** could be inverted and **R** could be processed by iteration.

### Cross-validation methods used for predictions of breeding values

We tested two different cross-validation scenarios, as follows: 165 cloned trees were divided into a training data set and a validation data set. In the first scenario, approximately 90% of the cloned trees (148) were sampled for the training set, either within each of the nine families or at random from the whole population without consideration of family origin. The remaining cloned trees were used for the validation (17 clones). In the second scenario, approximately 50% of cloned trees (84) were sampled either within family or randomly from the whole population for training, and the remaining cloned trees were used for validation (81 clones). For each scenario, six independent samples were analyzed. All the analyses were conducted with ASReml software (Gilmour *et al.* 2009). The SEs of breeding value predictions for each type of relationship matrix were averaged across the six independent validation samples for purposes of comparing the accuracy of predictions.

We examined the correlation between predicted breeding values of 165 clones (EBV1) from the ABLUP and GBLUP regression methods. To avoid bias in the EBV1 for the cloned trees, the pedigree data were omitted, and EBV1 were calculated based only on the phenotypes of the 16 to 50 ramets (genetically identical copies) of each cloned tree. We report the accuracy of a prediction as the correlation between the predicted genetic values (GEBV) from the cross-validation and the nonpedigree-based estimated breeding value (EBV1), which will determine the potential gain using markers (Meuwissen and Goddard 2010). We produced scatter plots and product-moment correlation coefficients between GEBVb and EBV1 values for all the validation populations (50% and 10% of cloned trees sampled within family). Also, to compare the predictive power of GBLUP and ABLUP on the validation sets, the correlation and scatter plots between GEBV and EBV2 values for the same replicates were produced.

## Results and Discussion

We used different methods to impute missing genotypes for calculation of genomic relationships among trees to use in genomic estimated breeding values. The results suggest that different methods of imputing missing genotypes did not have a noticeable effect on the accuracy of predictions in this study (Table 1). All four methods of imputing missing genotypes produced similar (0.71) accuracy values.

**Table 1:** Accuracy of genomic estimated breeding values based on different imputation methods

Method of Imputation	Accuracy of GEBV (by Eq. 1)
Stochastic, based on allele frequencies	0.70
Missing genotypes converted to zero	0.71
Continuous gene content, scaled	0.71
Continuous gene content, truncated	0.71

The different methods of imputing missing genotypes did not affect the prediction accuracies. GEBV, genomic estimated breeding value.

**Figure 1:**
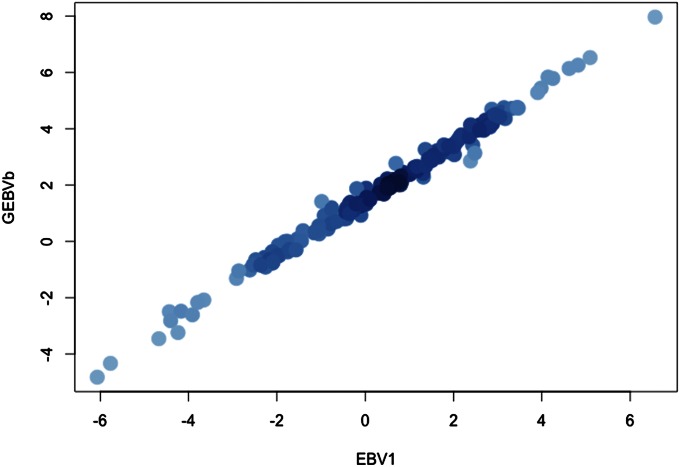
Scatter plot between predicted breeding values from ABLUP and GBLUP (regression method) for all 165 cloned trees. Predictions based on genomic relationships are highly correlated (*r* = 0.997) with the predictions based on nonpedigree-based analysis. Phenotypic data for all the genotyped trees (165) were included in both analyses (no subsampling for training and validation).

We compared predictions (EBV1) based on all the phenotypic data without the pedigree with GEBVs for all 165 cloned trees, using marker data to model realized relationships (Figure 1). The correlation between EBV1 and GEBV was almost perfect (0.997). This result indicates that family relationships are not affecting the estimates of breeding value, in contrast to the result reported for unbalanced datasets with little or no clonal replication of progeny genotypes (Garrick *et al.* 2009). The EBV1 values were therefore used as the true breeding values, the standard against which other estimated breeding values from the cross-validation studies were compared.

### Validation

Efficiency of markers in general is evaluated by a correlation between true and estimated breeding values (Meuwissen *et al.* 2001, Daetwyler *et al.* 2011). When 50% of individuals within family were sampled for validation, the correlations between the true breeding values coming from all the 165 clones (EBV1) and GEBV were 0.37 and 0.38 for allele frequency or regression method, respectively (Table 2). However, when a larger number of individuals (sampling 90% of trees within family) were used for training, we observed greater correlations of GEBV with the EBV1; *i.e.*, 0.52−0.55. The last two rows in Table 2 represent the correlations between EBV2 and GEBV. The EBV2 are predictions obtained for the validation set (no phenotypic data) from a traditional BLUP approach using the numerator relationship matrix. The correlation between GEBVa and estimated breeding values coming from only the cloned trees included in the validation set (EBV2) was 0.74 for 10% of sampled clones, and 0.69 for 50% of sampled clones. Similar high correlations were obtained between regression-based GBLUP (GEBVb) and EBV2 (Table 2). After comparing the four cross-validation methods used in this study, we found that predicting on 10% of the clones either sampled within family or at random was more accurate than predictions on 50% of the clones sampled for validation. This result could be due to a larger training model, where more trees were included to estimate the relationships between individuals.

**Table 2:** Results of cross-validation

Correlation	10% Within-Family Sampling for Validation	50% Within-Family Sampling for Validation
*r*_GEBVa-EBV1_	0.55	0.37
*r*_GEBVb-EBV1_	0.52	0.38
*r*_GEBVa-EBV2_	0.74	0.69
*r*_GEBVb-EBV2_	0.71	0.70

The first two rows show correlation coefficients of genomic estimated breeding values (GEBV) with estimates based only on phenotype (no pedigree, EBV1) for the whole population. The last two rows represent the correlations between GEBV and estimates from the validation set (pedigree is used without phenotypes, EBV2).

Breeding values based on realized genomic relationships obtained from SNP markers were as accurate as predictions based on the traditional pedigree-based genetic evaluation, based on comparison of SE estimates. The average SE for all 165 cloned trees using traditional genetic evaluation based on pedigree was 1.14, whereas the average SE for breeding values estimated using genomic relationships was about 0.71. This finding suggests that the marker genotypes used in construction of genomic relationship matrices, by either the allele-frequency method or the regression method, effectively reconstruct the family relationships in this structured population. The accuracy of prediction is higher for cross-validation scenarios that use larger training populations (90% of clones used for training *vs.* 50%), suggesting that a much larger training population (perhaps 1500−2000 trees) would result in even more accurate predictions of breeding value. Muir (2007) reported that the accuracy of GEBV for traits of low heritability can exceed that of the traditional BLUP approach if the training population is large enough.

### Mendelian sampling effect

**Figure 2:**
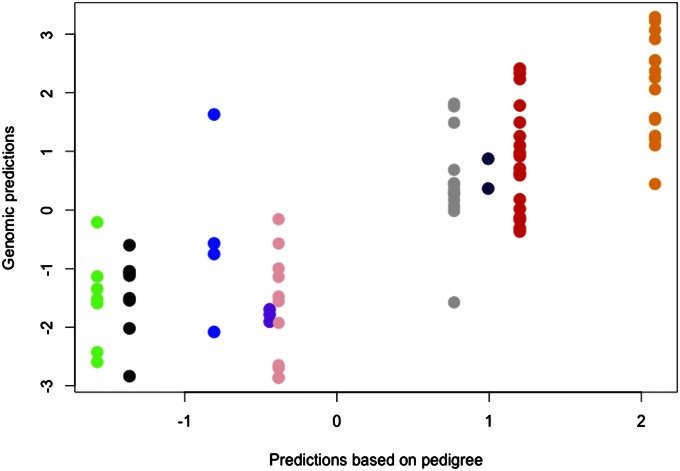
Scatter plots of GEBVa *vs.* EBV2 of individuals from different full-sib families used for cross-validation. The vertical axis is the genomic estimated breeding values based on allele frequency (GEBVa), and the horizontal axis is the breeding values based on pedigree derived A matrix (EBV2). As expected, without phenotype, the predicted breeding values (EBV2) of full-sibs are the same (mid-parent values). On the other hand we see segregation of full-sibs when GBLUP is used.

Scatter plots between GEBVa and EBV2 demonstrate the ability of markers (GBLUP) to capture Mendelian segregation within families (Figure 2). In the figure, we see full-sibs grouped for nine crosses. The traditional pedigree-based method for prediction of genetic merit of full-sibs with no phenotype (EBV2) produces the same prediction for every offspring within a full-sib family, the mid-parent breeding value (Falconer and Mackay 1996). In other words, when prediction is not based on phenotypic data, all full-sibs coming from a single cross have the same mid-parent breeding value, because Mendelian segregation is not modeled by the **A** matrix. However, when marker-derived genomic relationships are used for predictions, Mendelian segregation within full-sib families is modeled (Figure 2). Capturing the Mendelian sampling effect will have a significant impact on the accuracy of forward selection in tree breeding programs. Seedling genetic tests with a single observation per progeny genotype have been more common in conifer breeding programs than clonally replicated trials, and forward selection based on phenotypes of low heritability is a significant challenge. The use of a realized genomic relationship matrix could prove to be a powerful combination for identification of the individual with the best breeding value within each full-sib family.

### A *vs.* G matrices

When pedigree-based expected additive genetic covariances are used as in traditional genetic evaluation, the model assumes constant and categorical covariances between relatives. For example, full-sibs are expected to share 50% of alleles that are IBD, and thus the additive genetic covariance between full-sibs is assumed to always be 0.5 (Falconer and Mackay 1996). On the other hand, the realized genomic relationships among the same full-sibs can vary around a mean of 0.5. In this study the range of genetic covariance between full-sibs coming from one single full-sib family was 0.44−0.65. The realized relationship matrix indicates the specific relationship between each pair of individuals, estimated by marker tracking of the alleles they share that are IBD. Genomic relationships estimated from marker data provide more accurate estimates of genetic covariance between relatives, which in turn leads to more accurate predictions. We presume that the more accurate genetic covariances among relatives are essentially modeling which allele at a QTL was transmitted from the parent to the offspring, even though QTL are not explicitly modeled.

Our results are in agreement with the suggestions of Habier *et al.* (2007), in that we find molecular markers can capture additive genetic relationships even when linkage disequilibrium (LD) is low, and can generate GEBV with accuracy different from zero. Using simulations, they demonstrated that with a significant level of LD in the population, the accuracy would be expected to be greater than with low levels of LD. Furthermore, they showed that the simulated accuracies over generations declined as the LD decays with additional recombination. LD has been reported to be low in pine populations (Brown *et al.* 2004), suggesting that any LD contributing to the accuracy of GBLUP in our analysis is likely to have arisen due to sampling effects related to the small number of founding parents of our experimental population (Grattapaglia and Resende 2011). This finding suggests that the potential advantage of GEBV needs to be estimated for each breeding population based on the contribution from LD to the accuracy of the predictions, and that phenotypic measurements and genotyping to model the genetic control of phenotypic variation may be required in every generation to maintain the predictive accuracy of genomic breeding models. Use of GBLUP methods to select individuals from the same generation as the training population, as modeled by our cross-validation studies, is likely to be more accurate than using GBLUP to predict the value of progeny of the training population. If LD arises through the sampling effect of small founding population size, recombination will decrease the LD from one generation to the next.

### G matrices from regression *vs.* allele frequency

We used two methods for calculating genomic relationships (allele frequencies and regression). In general, they were comparable in the accuracy of the predicted genomic breeding values. The main advantage of the method based on allele frequencies is that the observed minor allele frequencies were included to scale the **G** matrix, with no consideration of inbreeding or selection. The markers used in this study represent a very small fraction of the genome, and probably explained a very small fraction of phenotypic variation. It would be possible to use the average allele frequencies from the base population, as provided by a larger set of families and parents included in previous mating designs. The genomic relationships should be estimated using the allele frequencies from the unselected base population. The regression based method employs the marker effects as dependent variable, and the mean homozygosity is adjusted by regressing the markers on **A** (dependent variable). This method was efficient for control of bias in the predictions, because it includes a matrix of residuals of the differences between true and expected fraction of alleles shared in common, as well as a measurement residual to account for the fact that the genotyped markers represent only a subset of the entire individual tree genome. When allele frequencies in the base population are different from 0.5, rare alleles contribute more to the genetic resemblance between individuals than common alleles (Forni *et al.* 2011). Those authors compared different methods to calculate **G** matrices and reported similar high correlations (~0.98) between methods, and small differences in the ranking of livestock individuals with different genomic matrices. They suggested that modifications to the **G** matrix could be important for different species or different populations. For example, the **G** matrix can be normalized to have average diagonal coefficients equal to 1 to assure compatibility with an **A** matrix, when either the average inbreeding or the number of generations are low, as is the case in most forest tree breeding populations.

Based on minor allele frequency, a subset of markers can be explored to estimate the realized genomic relationship matrix. For example, marker loci should be chosen so that all parents are heterozygous for at least a few rare minor alleles. Selecting loci with low minor allele frequency can create problems with singularities in the genotype matrix, if there are many homozygous genotypes across the individuals. Forni *et al.* (2011) gave a possible solution, using weighting of the genomic relationship matrix by the A matrix to eliminate singular matrices, if the number of loci is limited or two individuals have identical genotypes across all markers.

If markers are assumed to contribute differently to the genetic variance, an alternative approach is the use of non-linear methods for prediction of marker effects (Meuwissen *et al.* 2001; Habier *et al.* 2007; VanRaden 2008). Bayesian methods are a common alternative, due to the fact that small estimated effects can be regressed toward zero; larger estimated effects can be regressed less, to account for a non-normal prior distribution. The different methods for generating **G** matrices, compared with Bayesian methods to estimate marker effects, resulted in a slightly greater accuracy of nonlinear models in some simulations (VanRaden 2008). Other authors (*e.g.*, Habier *et al.* 2007) have reported that accuracies from Bayesian methods were comparable with the accuracy of Ridge regression if many different loci contribute equally to the simulated phenotypes, while Bayesian methods give better performance if a few loci account for much of the phenotypic variation.

### Application of GBLUP in forest tree breeding

Genomic BLUP has some advantages over genome-wide predictions of breeding values using specialty software such as GS3, developed by Legarra and Misztal (2008). The GBLUP procedure simply requires replacing the numerator relationship matrix with the realized genomic relationship matrix, so it is straightforward to model complex variance-covariance structures, such as genotype by environment interactions in plant breeding. There are established procedures and software, such as ASReml (Gilmour *et al.* 2009), to run such models. Genomic BLUP is simply an analog of traditional genetic evaluation based on pedigree and phenotype with possibly a few additional factors in mixed models, and is simpler than solving large numbers of equations to model marker additive and dominance effects simultaneously (Zapata-Valenzuela *et al.* 2012).

Another advantage of using a genomic relationship matrix over fitting thousands of markers simultaneously to estimate breeding values is that GBLUP requires a much smaller number of markers to construct realized genomic relationships. Grattapaglia and Resende (2011) suggested that for forest trees a density of 10−20 markers per centimorgan would be necessary for genomic selection, depending on the degree of LD in the training and selection populations. Given the large genome size and relatively low population-wide LD of most forest trees, particularly conifers such as loblolly pine, hundreds of thousands of markers might be needed to produce accuracies similar to those obtained from classical evaluation. High density genotyping would require cost effective, repeatable genotyping platforms for routine application of genomic selection in forest trees. GBLUP is an appealing approach for forest trees to overcome high-density genotyping costs, and is an alternative to pedigree construction as suggested by El-Kassaby *et al.* (2011).

GBLUP is expected to play a major role in forest trees compared with pedigree reconstruction or genomic selection because it allows modeling the Mendelian segregation effect, it allows straightforward modeling of experimental design factors, and it requires a fraction of the number of markers required for genomic selection. The computation required is simple to implement. Predictions are less biased than those based on average relationship matrices (Legarra and Misztal 2008). It is also a way to generalize to complex models such as random regression or multi-trait analyses.

To our knowledge there are no studies in forest trees on using genomic relationship matrices for predictions. The accuracy of genomic estimated breeding values based on genomic relationship matrices would be lower in cases of strong genotype by environment interactions, but the same would be true of pedigree-based BLUP also. Forest trees are typically progeny tested across diverse environments. The linkage phase of markers and beneficial alleles at trait loci could be different in environments with contrasting climatic and edaphic conditions, and prediction models developed in one environment might not have the same predictive power in a different environment. Resende *et al.* (2012) suggested that a prediction model based on a training population grown in one environment had considerably lower accuracy in other environments, for a cloned loblolly pine experimental population.

Two hypotheses were tested in this experiment: (1) using genomic relationships derived from SNP marker genotypes could be an efficient approach to increase the reliability of breeding value predictions of forest trees, and (2) two different methods of estimating realized genomic relationships from marker genotypes yield equivalent accuracy of breeding value predictions. We found that the predictions based on genomic relationships derived from markers (GBLUP) were as accurate as traditional genetic evaluation based on an average relationship matrix derived from the pedigree. Second, our results showed that genomic relationships generated either by the allele frequency method or the regression method did not differ in the accuracy of genomic estimated breeding values. Realized genomic relationships captured Mendelian segregation among full-sib individuals, even in the absence of phenotypic data, which was not the case with the traditional genetic evaluation. In practical terms, using a genomic relationship matrix is no more difficult than using the traditional numerator relationship matrix derived from a pedigree. The GBLUP model could use raw measurement data rather than deregressed breeding values as phenotypes; it incorporates fixed effects of common environments and genotype by environment (G×E) interactions; and it allows spatial analysis and other more complex variance−covariance structures. These characteristics will allow easy implementation of future applications of GBLUP using standard software available for linear mixed model analyses.

## Supplementary Material

Supporting Information
