# Supplementary material for: Genomic Estimated Breeding Values Using Genomic Relationship Matrices in a Cloned Population of Loblolly Pine
Source: G3 (Bethesda). 2013 May 1;3(5):909–16. doi: 10.1534/g3.113.005975 (PMC3656736; doi:10.1534/g3.113.005975)
Supplement: Supporting Information [file supp_3_5_909__index.html]

Genomic Estimated Breeding Values Using Genomic Relationship Matrices in a Cloned Population of Loblolly Pine — Genomic Estimated Breeding Values Using Genomic Relationship Matrices in a Cloned Population of Loblolly Pine — Supporting Information 

# Genomic Estimated Breeding Values Using Genomic Relationship Matrices in a Cloned Population of Loblolly Pine

## Supporting Information for Zapata-Valenzuela *et al.*, 2013

**Files in this Data Supplement:**

- File S1 - Genotypes (.csv, 1 MB)
- File S2 - Phenotypes (.csv, 318 KB)
